# Memoir on the Morbific Elongation of the Tongue out of the Mouth

**Published:** 1801-11

**Authors:** Pierre Lassus, G. Bellamy


### *Memoir on the morbific Elongation of the Tongue out of the Mouth.* By PIERRE LASSUS.

[ Continued from p. 333—357 of our laſt. ]

WE read in the Actes Litteraires de Suède,* that the Society of Medicine at Stockholm, aſſembled in 1695, to examine the diſeaſe of a girl ten years old, who, from her birth, was disfigured by the protruſion of the extremity of the tongue: this organ was two inches thick, and hung on the chin the length of four inches: the under jaw was depreſſed by the weight of this tumor; the inciſive, canine, and ſmall molar teeth were turned forwards. This girl could neither ſhut her mouth, ſpeak diſtinctly, or retain her ſaliva, which flowed out continually in very great quantity; deglutition was troubleſome. This examination made, the conſulters decided, that the protuberant extremity of the tongue ought to be cut off; and Hoffman, ſurgeon of Stockholm, took upon himſelf the operation: it was done in ſuch a manner as not to give a favourable idea of Swediſh ſurgery in the laſt century. † The ſuperfluous portion of the tongue being cut off, it was weighed, and found to weigh three ounces. The hæmorrhage, although not conſiderable, was ſtopt by the application of the actual cautery. It is ſaid that this girl was perfectly cured in about three weeks; ſhe could afterwards ſpeak freely, and ſwallow with facility; the inferior lip, which from her birth had been turned by the tongue upon her chin, reſumed its natural ſtate: In fine, the author of this obſervation terminates his relation by an addreſs to the Maſters of the Art, and inviting them to turn to the profit of ſcience this operation, or to correct it if poſſible.‡ We ſee in a moment, that there is a great deal to correct in this manner of proceeding, for the cure of the diſeaſe in queſtion. It is alſo without neceſſity that Claudini, phyſician, of Bologna, in ſpeaking of a girl who from her birth had the tongue out of the mouth, adviſed, to obtain a radical cure, the cutting off this organ.§ All the authors of whom we have juſt ſpoken, knew

---

* Acta Litteraria & Scientiarum Sueciæ, Anno 1732, tom. 3. p. 1.

† The tongue being fixed on a kind of wide and hollow ſpatula, the protuberant extremity of this organ was cut off with a chizel, very much like that which ſculptors and ſtone-cutters uſe. Ferrum idipſum ad inſtar Ferri quo utuntur Sculptores & Lapicidæ. (P. 3.)

‡ Veſtrum eſt artis ſalutaris Chirurgiæ Magiſtri nobile hoc inventum in veſtros uſus vertere, vel ſi poteritis, illud emendare. (Ibid. p. 3.)

§ Quod ſi ſpectemus auctam linguæ magnitudinem plus juſto, vel malum hoc knew neither the nature or character of this disease, which is curable by the most simple means, even when it is inveterate. It is a fact that the tumor has not always the same volume, nor the same elongation. Those on whom this disease has existed for many years, have made this remark of it themselves: it has not escaped the greatest number of observers, but they knew not how to take advantage of the fact, or to seize the indication that this variable state of the affected part presented. The disease was never seen to be cured spontaneously; but the tongue has been seen, after being excessively protruded out of the mouth, to return partially into it, at least for some time, to detumify, and to render by this retrocession, more or less durable, the disease a little easier to be supported. It would be to deceive oneself to take the tumefaction of the tongue for the disease itself; that is, the effect of it, and not the cause. It may, to a certain point, be an obstacle to the reduction of the part, but it is so easy to diminish its size, to obtain its reduction afterwards, that it is astonishing that the most learned persons of the art have deceived themselves on this head. The inspection of the disease sufficiently indicates that for its cure the tumefied and elongated extremity of the tongue ought not to be cut off, but that the detumescence ought first to be effected, and then the reduction. This will be proved by the following observations: A few years since I was consulted for a child, about seven or eight days old, that constantly held the point of its tongue out of the mouth about the length of a finger's breadth. The fear that this vicious affection, which manifested itself immediately after birth, and which insensibly increased, might become still more considerable, had determined the parents to take my advice; the child was in other respects very well, but seized the nurse's breast with difficulty. I examined the tongue with attention; it was scarcely thicker or wider than in its natural state, it had no defect in original conformation, and it was easy for me to remit it into its natural place, which it immediately left as soon as I ceased to support it there. Instructed by this examination, I conceived the possibility of opposing, at the same time, the progress of the disease, and even of radically curing it, in supporting the tongue when reduced, and by opposing its exit; therefore I reduced it a second time, after having stimulated its point with a little crude alum in powder, and I applied under

---

hoc est nativum quale ego vidi in puella quæ linguam exertam gestabat, cui ea sensim etiam a primordio sui ortus fuit adaucta, lingua plus justo aucta incidatur. (Julii Cæsaris Claudini Empirica rationalis. Bononiæ, 1653, in fol. lib. iii. cap. 6. p. 603.)

der the chin a narrow and longitudinal comprefs, in the fhape of a fling, the ends of which I fixed on the top of the head, fo as to keep the jaws exactly clofed; I recommended that the child fhould no longer fuck, but to nourifh it with a mixture of cow's milk and barley water, to be taken by a fpoon, to the end that the tongue, by this contrivance, might excite, at leaft during deglutition, an inverfe action to that it performed in fuction, when the child fucked its nurfe. The parents took care not to move the apparatus which I had applied, but when it was neceffary to give the child drink, and to re-apply it immediately afterwards. This method, fo fimple and fo natural, fufficed to produce, in the fpace of fifteen days, a radical cure, and to permit the child afterwards to refume, without inconvenience, its nurfe's breaft. Curious to know what might be the caufe of this morbific difpofition of the tongue, I learnt from the midwife that the child came into the world by a face prefentation, with its face very red, the mouth open, and full of froth; that an abundant falivation appeared in the beginning, and that the accouchement had been long and difficult. From all thefe details it appeared to me plain that the protrufion of the tongue, which fhewed itfelf immediately after birth, was the confequence of a convulfive ftate, or of a kind of fuffocation, very like to that which fome epileptic perfons experience, who during the fit have the tongue exceedingly elongated out of the mouth. A little while after, I was confulted for a little boy, fix years old, who had a voluminous tongue, and out of the mouth about the length of three fingers breadth. This deformity had fubfifted more than three years. The parents, not knowing the caufe of it, attributed it to a bad habit which the child had contracted. However, the tongue was too much tumefied to be reduced and maintained in its natural place. The indication to be followed appeared to be, firft, to effect the detumefcence of the organ by the application of leeches. In fact, this local bleeding diminifhed its fize, and afterwards facilitated its replacement in the mouth. To keep it there, I caufed the jaws to be brought one againft the other by the application of a bandage under the chin, which was attached to the child's cap on the top of the head; I recommended this bandage to be kept on night and day, and only to be removed at meal times. By conftantly keeping the jaws together, to prevent the exit of the tongue, it gradually regained its natural fize, and in the fpace of a month the child was perfectly cured of its deformity. The incifive and canine teeth of the lower jaw were thrown forwards, as we have feen in the preceding obfervations; the inferior part of the tongue was flightly ulcerated by rubbing

 againft againſt the teeth, whilſt its ſuperior part was abſolutely dried up by continual expoſure to the air. The effuſion of ſaliva was not conſiderable, deglutition pretty eaſy, but ſpeech thick, and the ſound of the voice harſh. During the firſt eight days of the treatment, I perceived that the child could keep the tongue very well in the mouth, but that it came out immediately he opened it and ſpoke. I recommended ſilence; and ſaw clearly, that it was to the conſtant proximity of the jaws that the cure of this diſeaſe was to be attributed.

G. BELLAMY.

