# Errata

**Published:** 1802-10-01

**Authors:** 


——— p. 435, l. 25, *read* we SHALL ſee in a moment.

——— p. 437, l. 22, *read* OR IF YOU LIKE, of a kind of, &c.

